# Diagnosis delay a family of Galloway-Mowat Syndrome caused by a classical splicing mutation of *Lage3*

**DOI:** 10.1186/s12882-022-03000-5

**Published:** 2023-02-08

**Authors:** Yan Chen, Yan Yang, Yang Yang, Jia Rao, Haitao Bai

**Affiliations:** 1 Department of Pediatrics, The First Affiliated Hospital of Xiamen University, Xiamen, Fujian 361000 China; 2 Pediatric Key Laboratory of Xiamen, Xiamen, Fujian 361000 China; 3 Institute of Pediatrics School of Medicine, Xiamen University, Xiamen, Fujian 361000 China; 4 Department of Nephrology, Children’s Hospital of Fudan University, Shanghai, 201100 China

**Keywords:** Galloway-Mowat syndrome, LAGE3 gene, Early-onset nephrotic syndrome, Focal segmental glomerulosclerosis, Steroid-resistant nephrotic syndrome

## Abstract

**Background:**

Galloway-Mowat syndrome (GAMOS) is a group of rare hereditary diseases by the combination of early onset steroid-resistant nephrotic syndrome (SRNS) and microcephaly with brain anomalies caused by *WDR73*, *LAGE3*, *OSGEP*, *TP53RK*, *TPRKB*, *GON7*, *WDR4* or *NUP133* mutations.

**Case presentation:**

We present the clinical and genetic features of a two-year-old boy with early nephrotic syndrome, microcephaly, growth retardation hypotonia and hypothyroidism. Genetic testing showed the presence of a canonical-splice mutation in the LAGE3 gene (NM_006014: c.188 + 1C > T). A total of nine female members of the family carried the variant. Seven male members died prematurely, and three of them suffered from nephrotic syndrome, which is consistent with the x-linked gene map of the disease. The overall symptoms of the disease due to the *LAGE3* mutation were mild compared to other pathogenic genes.

**Conclusion:**

As far as we know, this is the largest family case of GAMOS2 caused by LAGE3 mutation found so far. We also compared other subtypes of GAMOS. Due to the heterogeneity of the renal phenotype, regular proteinuria screening is recommended for all patients diagnosed with GAMOS.

## Background

Galloway-Mowat syndrome (GAMOS) is a rare genetic disease that has these typical clinical manifestations, such as microcephaly, central nervous system abnormalities, and early-onset hormone-resistant nephrotic syndrome (steroid-resistant nephrotic syndrome, SRNS). In recent years, with the deepening of disease research, 54 GAMOS families with genetic abnormalities have been reported globally. In addition to kidney and neurological symptoms, clinical phenotypes have been found to include intrauterine growth retardation, optic nerve atrophy, and nystagmus, hearing impairment, craniofacial deformities, abnormal skeletal development of extremities, congenital heart disease and hypothyroidism. 67% of cases died before the age of three [1]. We report on the probands and their families of Galloway-Mowat syndrome caused by a classic splicing mutation in the LAGE3 (L antigen family member3) gene.

## Case presentation

**Fig. 1:**
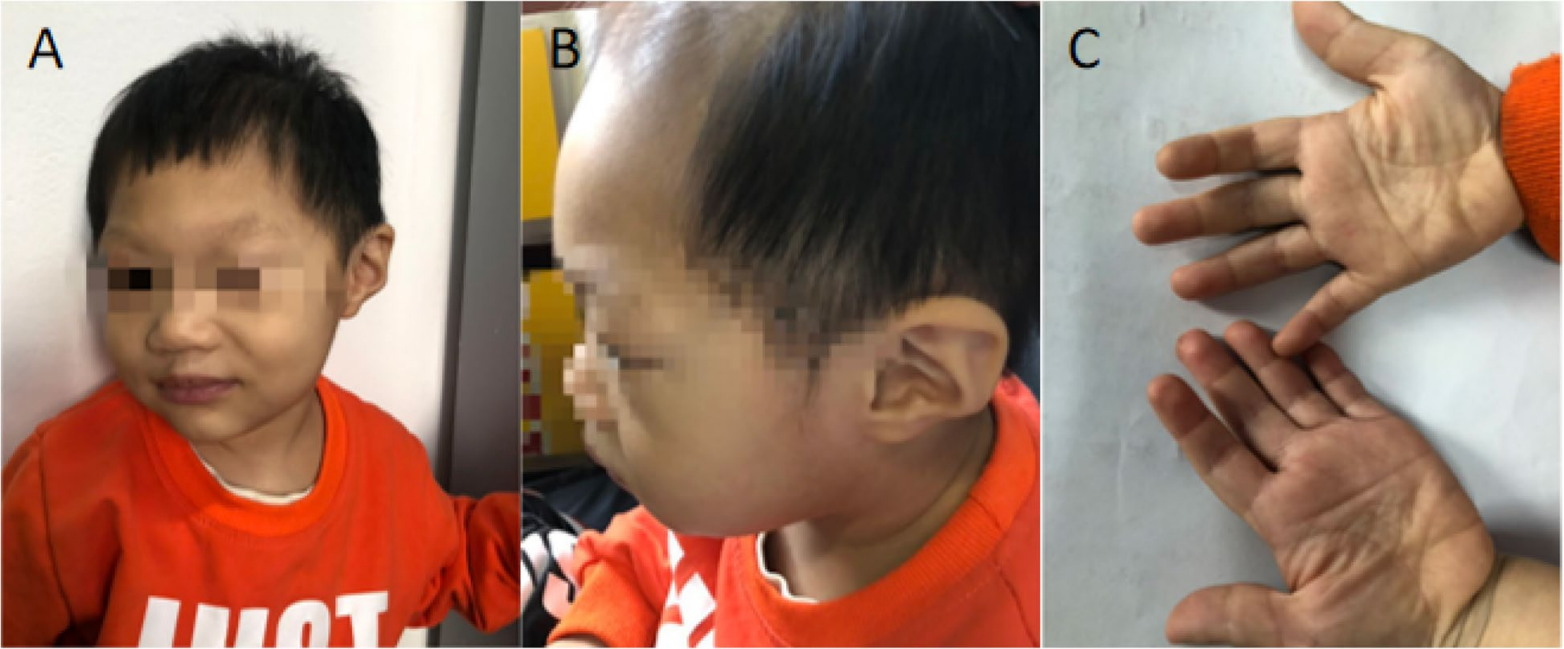
The image of the proband. **A** and **B** The forehead of the proband is narrow and inclined. **C** All fingers are short and stubby. There are simian lines on both hands

A 2-year-old male patient presented to our hospital in September 2019 due to "abnormal urine test found for 11 days". The physical examination of the patient 11 days before admission was found to be positive for urine protein, and the 24-h urine protein was 784 mg. The patient showed growth retardation and delayed development in the past. He raised his head at 6 months, sat at 10 months, walked at 1 year and 8 months, and is now 2 years old with unstable gait and can speak individual monosyllable words. The father suffers from ankylosing spondylitis, the mother has a positive urine protein, and they are not consanguineous. The patient has a sibling who is in good health. Physical examination of the patient on admission: body temperature 36.5 ℃, respiratory rate 27 breaths/min, heart rate 115 beats/min, head circumference 45.3 cm, weight 11.2 kg (10-25th percentile), height 80 cm (below the 3^rd^ percetile);.There are simian lines on both hands.All fingers are short and stubby.The forehead is narrow and slanted. The patient's cardiopulmonary examination is not special. He has weak muscle tone (Fig. 1).Main laboratory results showed on Table 1. Immune set, ANCA-related antibodies, anti-glomerular basement membrane antibodies, four items before blood transfusion, urinary system ultrasound, electroencephalogram, and brainstem auditory evoked potentials were all normal.

**Table 1:** Main laboratory examination indexes of the proband

	index	analytical finding	reference range
Urinalysis	pH value	7.0	5.0–9.0
	SG urine protein	1.021 2 +	1.005–1.030 negative
	urinary occult blood	negative	negative
	the ratio of urineprotein/creatinine albumin	2.018 29.4 g/L	< 0.03 35-50 g/L
Biochemical results	urea nitrogen Creatinine eGFR triglycerides cholesterol uric acid	3.18 mmol/L 29umol/L > 120 ml/min.173m^2^ 3.71 mmol/L 7.52 mmol/L 257 mmol/L	2.6–7.5 mmol/L 41-81umol/L 0.4–1.82 mmol/L 3.1–5.2 mmol/L 155-357 mmol/L
Thyroid function	thyroid-stimulating hormone free triiodothyronine free thyroxine	15.244mIU/L 0.89pmo/L 3.46 pmol/L	0.64–6.27mIU/L 5.1–7.4 pmol/L 11.1–18.1 pmol/L

**Fig. 2:**
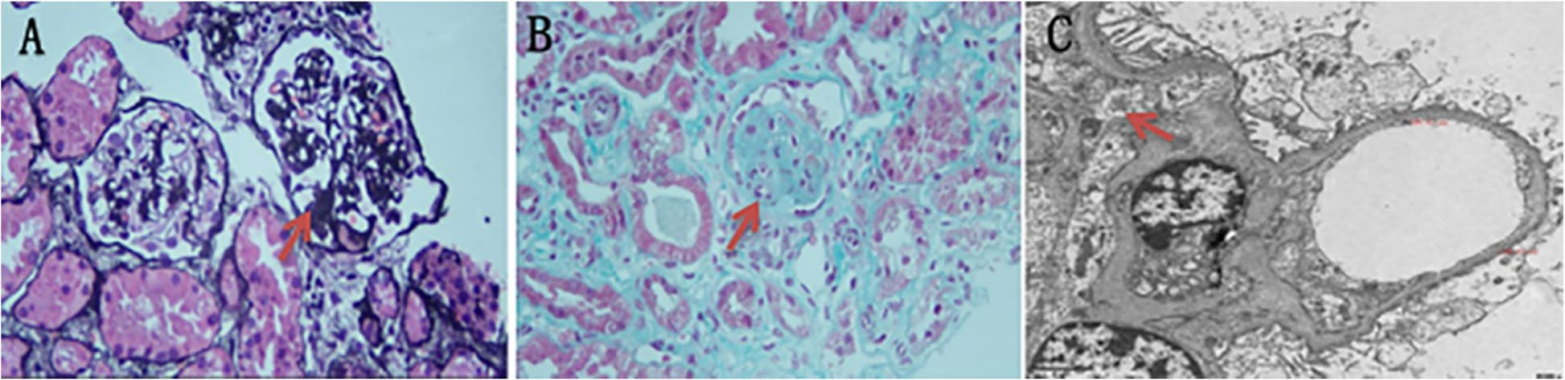
The image of renal biopsy from the proband. **A** PASM staining. **B** MASSON staining glomerular sclerosis. **C** Foot process fusion under electron microscope)

After the child was admitted to the hospital, the examination was completed, and levothyroxine tablets were taken orally to treat hypothyroidism. The proband undergoes kidney biopsy under general anesthesia. The pathological results suggested that 2/25 glomeruli were focal segmental sclerosis. Immunofluorescence showed no deposition of immune complexes. Under the electron microscope, the glomerular foot processes were diffusely fused with microvilli, and no precise electron dense deposits were seen. The patient was diagnosed with focal segmental glomerulosclerosis (FSGS) (Fig. 2).

**Fig. 3:**
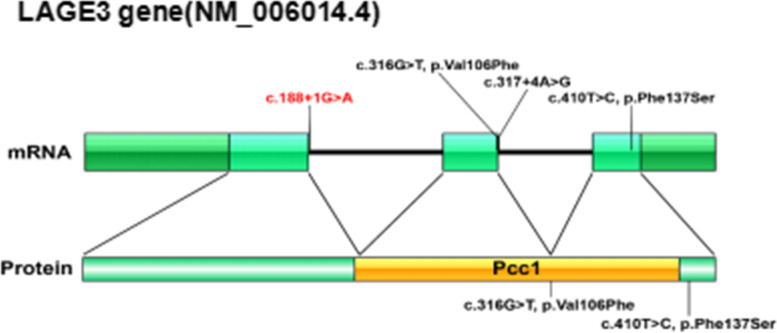
Schematic diagram of *LAGE3* gene structure and protein domain

Combined with the child's medical history and family history, genetically related nephrotic syndrome may be considered. Further investigation of the family history revealed that many male members of the family died prematurely with unknown etiology. One of the male members who died (III-3) had abnormal urine test. The other three male members (IV-5, IV-6 and IV-9) suffered from nephrotic syndrome and died at the age of 3, 5 and 8. In order to clarify the cause, after medical ethics review and the parents of the child signed an informed consent form, 2 mL of the peripheral blood samples of the child and the parents were collected for whole-exome genome sequencing. The results showed that the LAGE3 gene on the X chromosome of the child had a classical splicing site (NM_006014.4:c.188 + 1G > A), which was rated as pathogenic according to ACMG guidelines. This variant is a known pathogenic variant [1]. At present, the professional version of HGMD data only includes 4 variants of the *LAGE3* gene, including one classic splice site (c.188 + 1G > A, c.317 + 4A > G), and two missense variant sites (c.316G). > T, c.410 T > C). The *LAGE3* gene includes 3 exons. The encoded protein has a functional domain Pcc1 (transcription factor Pcc1). Pcc1 is a transcription factor that functions in regulating genes involved in cell cycle progression and polarised growth [2] (Fig. 3).

**Fig. 4:**
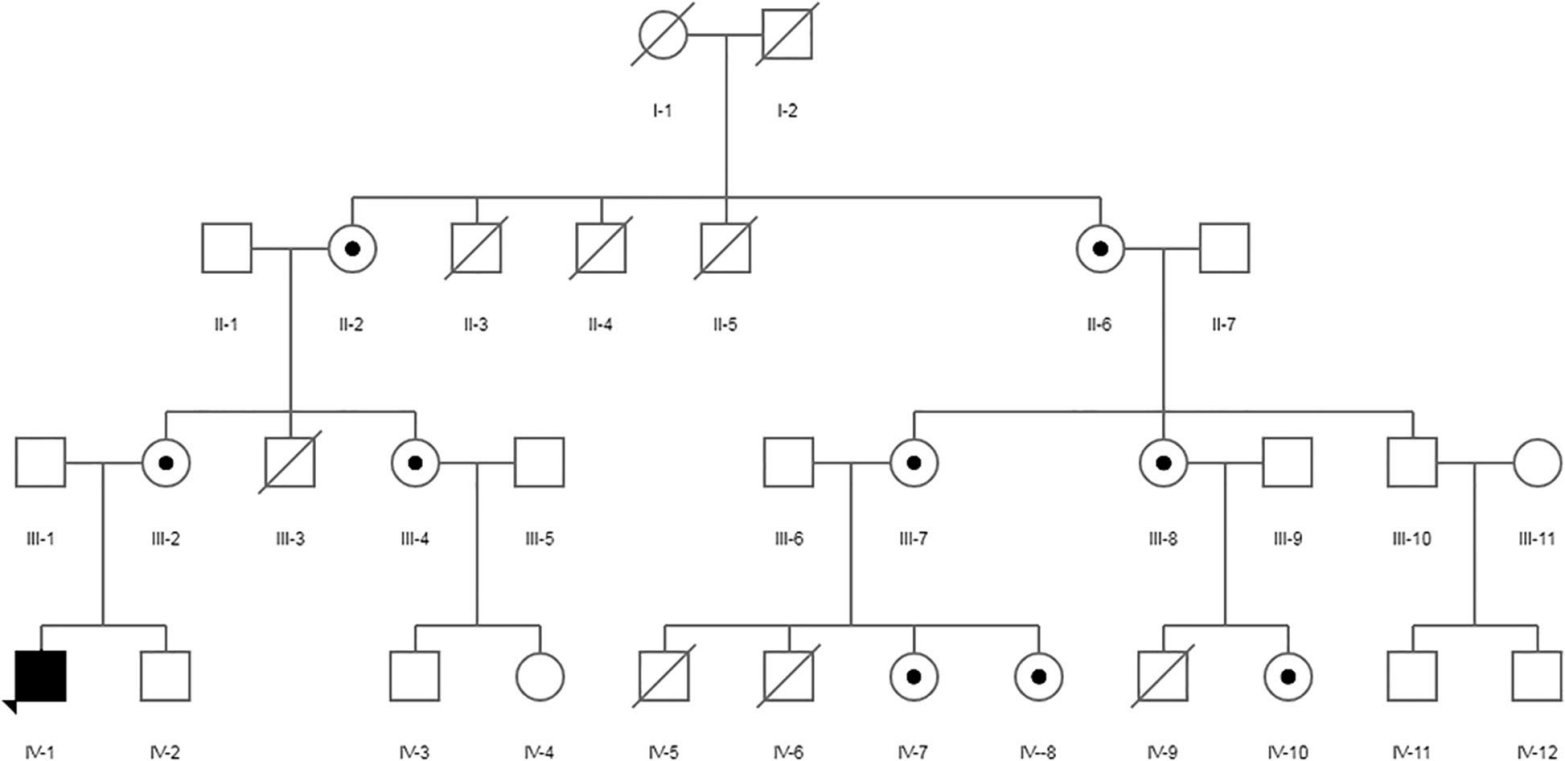
The pedigree of a family with Gallowy-Mowat syndrome caused by splicing mutation of LAGE3.  indicates female members carrying *LAGE3* mutation sites;  indicates as probands

The family members of the proband were verified by first-generation sequencing, and it was found that the mutation site was inherited from the mother, and the father and sibling brother were wild type. A total of 9 female members of the family (including the mother of the proband) all carried the mutation site, of which III-2, III-7 and III-8 were positive for urine protein, and IV-7, IV-8 and IV-10 for urine protein feminine. No urine routine was tested in II-2, II-6, and III-4. In addition, I-1 was due to Alzheimer's disease, and I-2 was due to brain tumor. II-3, II-4, and II-5 all died before the age of 5, and the specific cause was unknown. III-3 had abnormal urine test and died at the age of 5. IV-5, IV-6 and IV-9 all suffered from nephrotic syndrome and died at the age of 3, 5 and 8 years old respectively (Fig. 4).

The child was discharged after a complete examination. Regular follow-up visits are currently in the outpatient clinic. The child continued to take thyroxine tablets to treat hypothyroidism. At present, there is no abnormality in thyroid function of the patient.The case was not treated with glucocorticoid and immunosuppressant because of the lack of evidence-based medicine.Test and evaluate his urine output, blood pressure, and monitor urine routine, urine protein quantitative, kidney function and other related indicators.

## Discussion

Galloway-Mowat syndrome (GAMOS) was first reported in 1968, describing a pair of siblings suffering from the primary nephrotic syndrome-hiatal hernia-microcephaly triad [3].

Recent studies have revealed the important role of gene mutation in the pathogenesis of GAMOS. In 2014, Colin et al. [4] first reported that the loss of WDR73 (WD repeat domain 73) expression can lead to abnormal nuclei of glomerular podocytes, changes in microtubule networks, and cell viability. Braun et al. [1] found that the subunits coded by the four genes *LAGE3/OSGEP/TP53RK/TPRKB* constitute a highly conserved kinase-endopeptidase and other proteins of small size complex (KEOPS), which is one of the key factors in the pathogenesis of GAMOS. In animal experiments, *OSGEP*, *TPRK* or *LAGE3* mutations could lead to small head type in the early stage of zebrafish juvenile models. It also resulted in a significant reduction in the length of the cerebral cortex in mouse embryonic models. However, no kidney phenotype had been observed in the existing animal models, and it was speculated that the early lethality masked the appearance of the kidney phenotype. This indicated that the nervous system phenotype in GAMOS might appear earlier than the kidney involvement. Subsequent research by Arrondel.C [5] found that *GON7*, the fifth gene that constitutes KEOPS, combines with *LAGE3* and participates in cell stability and spatial structure arrangement.

The *WDR4* gene [6] is a newly discovered and consistent disease gene in recent years, which is located on chromosome 21 and has an autosomal recessive inheritance. In addition to microcephaly, general developmental delay and proteinuria phenotype, there are hypothyroidism. Growth hormone deficiency and other endocrine complications, but the degree of kidney involvement is not serious.

*NUP133* gene [7] mutations can also have clinical manifestations similar to GAMOS. The kidney pathology of 3 tested patients showed FSGS. The zebrafish model with this gene knocked out showed microcephaly, neurocytopenia, glomerular hypoplasia and Podocyte foot process fusion, which is similar to human GAMOS signs.

We summarized GAMOS1-5 types (Table 2) that have been reported to have gene mutations, and found several characteristics of the phenotype [3–17]. (1) High phenotypic heterogeneity. (2) Early onset of neurological symptoms. (3) The time and extent of the appearance of kidney phenotype are closely related to the lifespan of patients.

**Table 2:** Summary of genetics and clinical phenotypes of GAMOS 1–5 types

	GAMOS1	GAMOS2	GAMOS3	GAMOS4	GAMOS5
Gene	*WDR73* (15q24-q26)	*LAGE3* (Xq28)	*OSGEP* (14q11)	*TP53RK* (20q13.12)	*TPRKB* (2p13.1)
Number of reported families (proportion)	19/54(35.18%)	3/54(5.55%)	26/54(48.15%)	4/54(7.40%)	2/54(3.70%)
Inheritance	AR	XL	AR	AR	AR
Distribution area/ethnicity	Amish in North America; Arabs in Central Asia, West Asia, and North Africa	Not mentioned	Arab, Caucasians in North America, Taiwan, Spain, Pakistan	South Korea	not clear
kidney phenotype	Half of the combined NS or ESRD	Not mentioned	Early-onset SRNS and ESRD; Partial kidney tubule dysfunction; Hypomagnesemia and hypercalciuria	Extremely early NS and ESRD	Not mentioned
Time of appearance of kidney phenotype	9 months ~ 8 years	Not mentioned	2 days ~ 3 years and 11 months	1 day ~ 8 days	Not mentioned
kidney pathology	FSGS and DMS	FSGS and foot process fusion	MCD, FSGS, DMS and glomerular dysplasia, kidney tubular atrophy	FSGS with kidney tubular atrophy and interstitial fibrosis, glomerular dysplasia	FSGS and foot process fusion
Nervous system phenotype	80–90% of general developmental delay with hypotonia, microcephaly and extrapyramidal symptoms	Microcephaly	Similar to GAMOS1; refractory epilepsy	Microcephaly	Not mentioned
Nervous System Image	80% of microcephaly, some thin corpus callosum and cerebellum atrophy	Microcephaly with multiple cerebellar gyrus and diffuse cerebellar atrophy	Abnormal gyri and myelin development, part of cerebellum atrophy	Microcephaly, cerebellar atrophy and subarachnoid hemorrhage	Microcephaly and pachygyria
Ocular phenotype	Eye tremor and optic nerve atrophy	Not mentioned	Same as GAMOS1	Not mentioned	Not mentioned
Facial and bone abnormalities	Maxillofacial abnormalities (rough skin, thick eyebrows, broad nose, hirsutism and convex forehead)	Not mentioned	Maxillofacial abnormalities (narrow forehead, deep eye sockets, almond eyes, low ears, collapsed nose, small mandible and high palate), pectus excavatum	Maxillofacial abnormalities (similar to GAMOS3)	Not mentioned
Other cases	2/19 test tube baby; some close relatives married	Not mentioned	Aortic stenosis and high blood pressure; pyloric stenosis and hypothyroidism; some close relatives married	2/4 IVF; intrauterine growth retardation; hiatal hernia and gastric volvulus	Not mentioned
Average life span	1.5—28 years old	Not mentioned	2 months -8 years old	21 days—10 months	Not mentioned
Is it fatal in infancy	less	Not mentioned	The difference is large, most of them die within 1 year of age	Yes, die within 1 year of age	Not mentioned

*AR* recessive inheritance, *XL* X-linked inheritance, *NS* nephrotic syndrome, *ESRD* end stage kidney disease, *SRNS* steroid-resistant nephrotic syndrome, *FSGS* focal segmental glomerulosclerosis, *DMS* diffuse mesangial sclerosis, *MCD* minimal change disease, *IVF*, in vitro fertilization

We reported the GAMOS pedigree with the largest number of people discovered so far. The proband had a clear neurological and kidney phenotype, but the overall symptoms were mild. It was speculated that GAMOS2 caused by *LAGE3* mutations was a non-early lethal mutation based on family status. In addition to the proband, there were 7 male members of the family who had fatal manifestations in early childhood and childhood. Among them, 4 had abnormal urine tests, 3 had confirmed nephrotic syndrome, and 2 had been treated with glucocorticoids and other drugs. The cause of their death was ESRD and infection. The samples of the 9 female members submitted for examination all carried the same pathogenic site. Among them, 3 female members aged 30–39 years old had proteinuria, and 3 female members aged 6–10 years old showed no abnormalities in urine test. It was considered that the heterozygous kidney phenotype gradually appears with age. The pedigree analysis was consistent with the characteristics of X-linked inheritance caused by *LAGE3*. Consistent with the report of Braun [1], it was speculated that the fatal male cases in infancy and childhood in this family were hemizygous, and the kidney phenotype IV-7, IV-8 and IV-10 would gradually present with the increase of age.

The diagnosis time of this family is relatively delayed, and we believe that the main factors are as follows: (1) The patient's maternal family lives in remote rural areas, and the family members have a low overall cultural level and lack of knowledge of genetic diseases. There are neither local doctors with relevant expertise nor genetic testing available. (2) There are obvious individual differences in the severity of clinical phenotypes among different subtypes of GAMOS. For example, compared with the reported GAMOS3 and GAMOS4, IV-5, IV-6 and IV-9 in this family are not early fatal cases, which leads to insufficient attention of family members and doctors to the disease, and no further family history is traced.

## Conclusion

Kidney complications are the direct cause of most GAMOS deaths, except for sepsis, pneumonia, and severe electrolyte disturbances in a few cases. In view of the above, recommendations for screening and diagnosis of GAMOS include the following [6, 9, 17, 18]. (1) Prenatal screening and: For high-risk pregnant women with suspicious family history, in addition to genetic testing, prenatal ultrasound and fetal nervous system MRI can assist in the diagnosis in the second trimester. (2) Early diagnosis: In the infant stage, patients with abnormal urinary protein, obvious abnormal appearance, obvious family inheritance, and unexplained death of family members in childhood can be considered for genetic testing to determine or exclude the possibility of GAMOS. (3) Early intervention: kidney replacement therapy and supportive care including dialysis can significantly delay the progression of kidney complications. (4) Drug therapy: At present, no confirmed cases of GAMOS use immunosuppressive agents when the kidney phenotype appears. However, with the popularization of genetic testing, patients with single-gene hereditary nephropathy represented by GAMOS, even if the phenotype is SRNS, may have a partial response to immunosuppressive agents, which is expected to be achieved when combined with other therapies to control proteinuria (such as ACEI drugs) Clinical partial or even complete remission will create opportunities for individualized treatment in the future. (5) Close evaluation: Due to the heterogeneity of kidney phenotypes, it is recommended that all patients diagnosed with GAMOS be screened for proteinuria on a regular basis.

## Data Availability

The datasets used and/or analysed during the current study are available from the corresponding author on reasonable request.
